# First molecular description, phylogeny and genetic variation of *Taenia hydatigena* from Nigerian sheep and goats based on three mitochondrial genes

**DOI:** 10.1186/s13071-019-3780-5

**Published:** 2019-11-05

**Authors:** John A. Ohiolei, Joshua Luka, Guo-Qiang Zhu, Hong-Bin Yan, Li Li, Abdullahi A. Magaji, Mughees A. Alvi, Yan-Tao Wu, Jian-Qiu Li, Bao-Quan Fu, Wan-Zhong Jia

**Affiliations:** 1 State Key Laboratory of Veterinary Etiological Biology/National Professional Laboratory of Animal Hydatidosis/Key Laboratory of Veterinary Parasitology of Gansu Province, Lanzhou Veterinary Research Institute, Chinese Academy of Agricultural Sciences, Lanzhou, 730046 People’s Republic of China; 2 Department of Veterinary Parasitology and Entomology, Faculty of Veterinary Medicine, University of Maiduguri, Maiduguri, Nigeria; 3 Department of Veterinary Public Health and Preventive Medicine, Faculty of Veterinary Medicine, Usman Danfodiyo University, Sokoto, Nigeria

**Keywords:** Genetic variation, Haplotype, *Taenia hydatigena*, Sheep, Goat, Cysticercosis

## Abstract

**Background:**

Cysticercosis caused by the metacestode larval stage of *Taenia hydatigena* is a disease of veterinary and economic importance. A considerable level of genetic variation among isolates of different intermediate hosts and locations has been documented. Generally, data on the genetic population structure of *T. hydatigena* is scanty and lacking in Nigeria. Meanwhile, similar findings in other cestodes like *Echinococcus* spp. have been found to be of epidemiological importance. Our aim, therefore, was to characterize and compare the genetic diversity of *T. hydatigena* population in Nigeria based on three mitochondrial DNA markers as well as to assess the phylogenetic relationship with populations from other geographical regions.

**Methods:**

In the present study, we described the genetic variation and diversity of *T. hydatigena* isolates from Nigerian sheep and goats using three full-length mitochondrial genes: the cytochrome *c* oxidase subunit 1 (*cox*1), NADH dehydrogenase subunit 1 (*nad*1), and NADH dehydrogenase subunit 5 (*nad*5).

**Results:**

The median-joining network of concatenated *cox*1-*nad*1-*nad*5 sequences indicated that *T. hydatigena* metacestodes of sheep origin were genetically distinct from those obtained in goats and this was supported by high F_ST_ values of *nad*1, *cox*1, and concatenated *cox*1-*nad*1-*nad*5 sequences. Genetic variation was also found to be higher in isolates from goats than from sheep.

**Conclusions:**

To the best of our knowledge, the present study described the genetic variation of *T. hydatigena* population for the first time in Nigeria using full-length mitochondrial genes and suggests the existence of host-specific variants. The population indices of the different DNA markers suggest that analysis of long mitochondrial DNA fragments may provide more information on the molecular ecology of *T*. *hydatigena.* We recommend that future studies employ long mitochondrial DNA sequence in order to provide reliable data that would explain the extent of genetic variation in different hosts/locations and the biological and epidemiological significance.

## Background

Cestodes belonging to the genus *Taenia* infect a wide range of intermediate host species where the metacestode larval stage causes cysticercosis or coenurosis. A few members of this genus such as *Taenia solium*, *T. saginata* and *T. asiatica* are zoonotic and are responsible for taeniosis in humans while others are of veterinary importance.

The tapeworm *T. hydatigena* uses canids (primarily dogs) as definitive hosts. The metacestode larval stage of *T. hydatigena* infects mostly domestic animals such as goats, sheep and pigs, resulting in cysticercosis; an infection of veterinary importance that causes huge economic loses especially in livestock production as a result of mortality [1], condemnation of infected organs and carcasses, and the financial cost of diagnosis and inspection [2–5].

*Taenia hydatigena* has a global distribution with a prevalence range of 0.1–32.0%, differing between countries and hosts [4–6]. The epidemiology is such that prevalence is usually higher in sheep and goats in most African and European countries compared to countries in Asia [1, 4, 5], and higher in pigs in Asian and South American countries than in countries of other regions [4, 6, 7].

In Nigeria, cysticercosis due to *T. hydatigena* is poorly documented. However, existing reports have demonstrated high infection rates in livestock especially in sheep and goats [7–14]. Although the prevalence of cysticercosis seems to be quite high, knowledge of the molecular ecology and intraspecific variation of *T. hydatigena* species is lacking. Meanwhile, genetic variation in other cestodes, e.g. *Echinococcus granulosus* [15–19] has been found to affect host infectivity, epidemiology, as well as control strategies [20]. Similarly, studies on *T. saginata* and *T. solium* mitochondrial genome have also demonstrated intraspecific variation [21–24] suggested to influence the pathological presentations exhibited in different hosts [25].

Globally, there exists a dearth of data on the genetic variation of *T. hydatigena*. Although a few studies conducted mostly by using partial *cox*1 and *nad*1 mitochondrial gene sequences have reported considerable levels of genetic variation among *T. hydatigena* populations from different geographical regions and hosts [4, 26–29], the epidemiological implications if any are far from clear and more studies in this regard may further help us understand the genetic variation or genetic population structure of *T. hydatigena* that could provide better insight on the disease epidemiology.

Further, the limitation of partial gene sequences in inferring the phylogenetic status of taeniids has been largely emphasized for *Echinococcus* [30, 31]. Therefore, the available data based on partial gene sequences may have limitation in understanding the extent and significance of the genetic variation observed among *T. hydatigena*. Also, some studies have suggested that complete mitochondrial genes such as *nad*6, *nad*5, *atp*6, *nad*3 and *nad*2 may have some advantage over the conventional mitochondrial markers like *cox*1 and *nad*1 in investigating the molecular ecology of *Taenia* spp. [28, 32]. Therefore, in the present study, our aim was to identify and investigate the genetic variation of *T. hydatigena* population in Nigeria based on the *cox*1, *nad*1 and *nad*5 mitochondrial genes, compare the genetic variation between the genes, and infer the phylogenetic relationships with *T. hydatigena* populations from other geographical regions.

## Methods

Nigeria is a West African country with a population of over 180 million people. It is made up of 36 states and a Federal Capital Territory located in Abuja. These states are further grouped into six geopolitical zones (North-East, North-Central, North-West, South-East, South-South, and South-West) due to ethnicity and common ancestry. The vegetation cover is majorly rainforest in the south and savannah in the north. Borno State is located in the North-East zone of the country and home to about 4 million small ruminants (sheep and goats) [33] that are managed majorly by traditional method of livestock farming.

Isolates (*n* = 32) analyzed in this study were collected postmortem from goats (*n* = 24) and sheep (*n* = 8) in the months of November and December 2018 during routine examination at an abattoir in Maiduguri, Borno State, northeastern Nigeria (11°50′41″N, 13°8′89″E). All isolates were of liver origin.

### DNA extraction, amplification and sequencing

Genomic DNA was extracted from a cut piece of each cysticercus using Qiagen Blood and Tissue Kit (Qiagen, Hilden, Germany) according to the manufacturer’s instructions. PCR was performed in a final 25 µl reaction mixture containing 12.5 μl Premix Ex *Taq*™ version 2.0 (Takara Bio, Kusatsu, Japan), 10 pmol of each primer, 0.5 μl of genomic DNA extract (*c.*20–200 ng), and RNAse free water up to the final 25 μl volume. The reaction was as follows: initial denaturation at 95 °C for 5 min, followed by 35 cycles of denaturation at 95 °C for 30 s, annealing at 55 °C for 40 s, and elongation at 72 °C for 60–90 s and a final extension at 72 °C for 10 min. Amplicons were visualized by electrophoresis in 1.5% (w/v) agarose gels in 1× TAE (40 mM Tris-acetate, 2 mM EDTA, pH 8.5), stained with GelRed™, viewed under UV light and the products sequenced (Beijing Tsingke Biotechnology Co., Ltd., Beijing, China). Primers used for amplifying the complete *cox*1, *nad*1 and *nad*5 genes were designed by Primer Premier 5 software based on the full mitochondrial genome sequence of *T. hydatigena* (GenBank: NC_012896) (Table 1).

**Table 1:** Set of primers used for *Taenia hydatigena cox*1, *nad*1 and *nad*5 amplification (GenBank *Taenia hydatigena* reference: NC_012896)

Primer ID	Primer sequence (5′-3′)	Target gene	Product size (bp)	Mitochondrial region/position	GenBank ID	Reference
Th-nad1F	CGTTGGGTTTGCGTCTCAAAAATGG	*nad*1	1146	5055…6199	NC_012896	Present study
Th-nad1R	CCAAAGGTCCCCAAAACCATCATTC					
Th-nad5F	TAGGATTAATTTATGACTAGAGTCTCT	*nad*5	1927	11540…13466	NC_012896	Present study
Th-nad5R	CTTCTCTCAATCTACCACTAGAAGAGG					
Th-cox1F	CTGTTGGTTATGTTCGTAGTTTTT	*cox*1	2013	6531…8543	NC_012896	Present study
Th-cox1R	GGCAAATAAACCTAAAAACCCTACTC					

### Molecular analysis

Sequences were assembled stepwise with the help of DNAstar v7.1 program and Unipro UGENE v1.29.0 software while making sure that the overlap sequences were identical and then viewed manually for correction of any nucleotide misread followed by alignment in BioEdit v7.2.6 [34]. The identity of each isolate was confirmed with their nucleotide sequence in the GenBank database using the NCBI BLAST algorithm (https://blast.ncbi.nlm.nih.gov/Blast.cgi). Nucleotide and haplotype diversity indices were estimated in DnaSP v6 [35]. Median-joining network [36] was inferred based on the sequences of mitochondrial *cox*1, *nad*1, *nad*5, and concatenated *cox*1*-nad*1*-nad*5 genes using PopART (http://popart.otago.ac.nz). Pairwise nucleotide difference was calculated using MEGA 7 [37]. Population neutrality indices, Tajima’s D [38] and Fu’s Fs [39] were calculated using DnaSP v6 [35]. F_ST_ was calculated using the Arlequin 3.5.2.2 software package [40]. The Bayesian phylogenetic relationship of the Nigerian *T. hydatigena* isolates with other *Taenia* spp. was carried out based on *cox*1-*nad*1-*nad*5 (4083 bp) concatenated sequences with MrBayes v.3.1.2. The General Time Reversible model with a proportion of invariable sites and a gamma-shaped distribution of rates across sites (GTR + I + G) was used as the best-fit model of sequence evolution as determined by JModelTest [41]. Markov Chain Monte Carlo (MCMC) sampling was used to assess the posterior distribution of parameters with a chain length of 2,000,000 states, and 10% was discarded as ‘burn-in’. Parameters were logged every 1000 states. TreeView v.1.6.6. (http://taxonomy.zoology.gla.ac.uk/rod/treeview.html) was used to display the phylogenetic tree.

## Results

All 32 isolates (24 goats and 8 sheep) were identified as *T. hydatigena*. Overall, 29, 24 and 27 isolates were successfully amplified for *nad*1, *cox*1 and *nad*5, respectively. A BLAST search of the resulting nucleotide sequences showed > 99% similarity with *T. hydatigena* sequences deposited in the GenBank database.

### Sequence variation

**Fig. 1:**
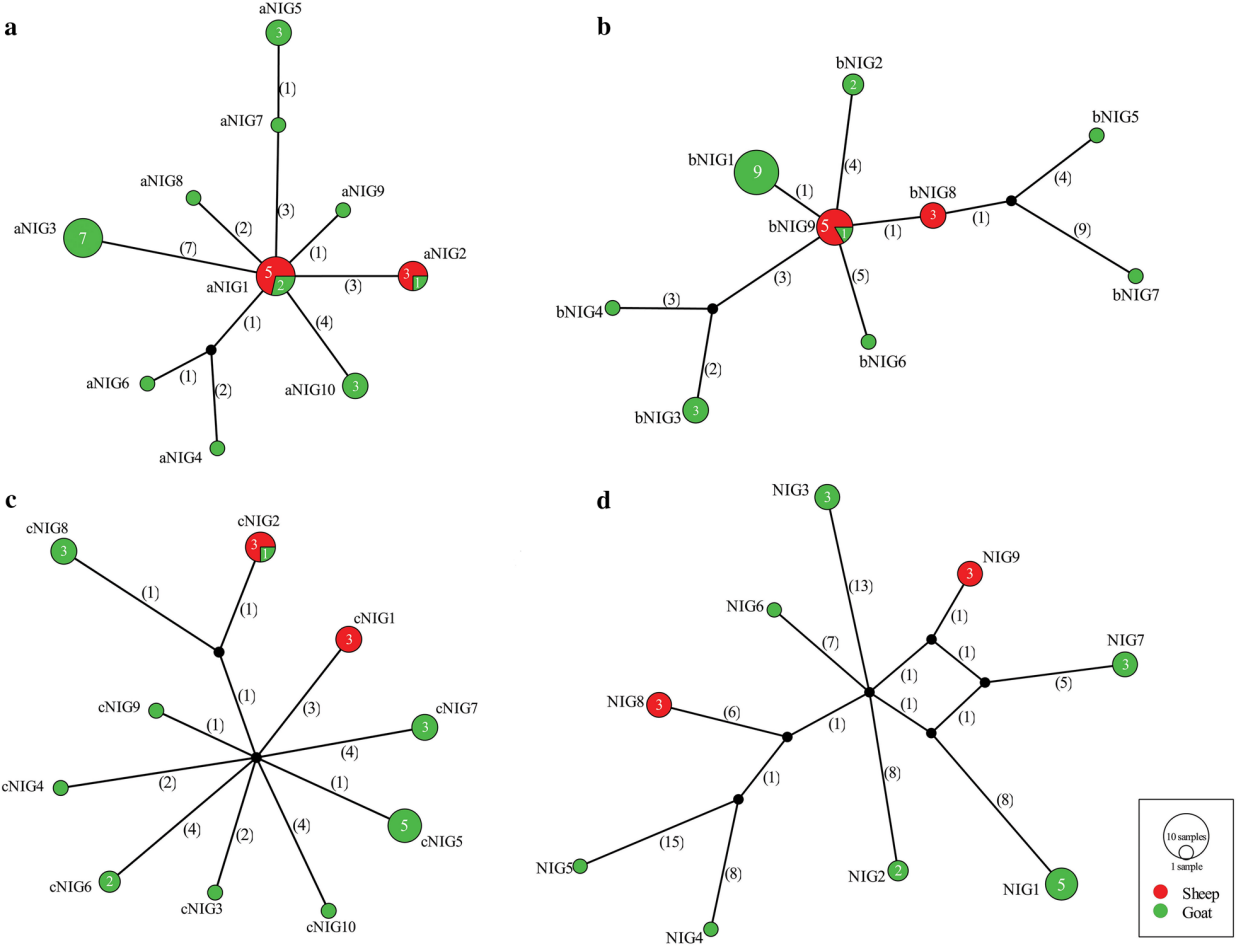
Median-joining networks of *Taenia hydatigena* isolates from Nigeria based on complete *cox*1 (**a**) *nad*1 (**b**) *nad*5 (**c**) and concatenated *cox*1-*nad*1-*nad*5 gene sequences (**d**). Circle sizes are proportional to the haplotype frequencies. Numbers in parentheses represent the number of mutations. Small black circles are median vectors (i.e. hypothetical haplotypes)

On analysis of the complete *cox*1 (1620 bp), *nad*1 (894 bp) and *nad*5 (1569 bp) mitochondrial nucleotide sequences, we observed 24, 24 and 33 polymorphic sites, respectively for each gene, of which 62.5% (15/24), 75% (18/24) and 36.36% (12/33) were parsimony informative, respectively. The number of haplotypes (Hap) observed was 10, 10, 9 and 9 for *nad*1, *cox*1, *nad*5 and *cox*1-*nad*1-*nad*5 sequences, respectively (Table 2). According to the median-joining network, the haplotype aNIG1 appeared centralized for *nad*1 gene sequences and constituted 24.13% (7/29) of the total population with 1–7 mutational differences from the other haplotypes (Fig. 1a). For *cox*1 network, no central haplotype was observed while for *nad*5, bNIG9 was at the center of the network with 1–10 mutational steps from the other haplotypes constituting 22.22% (6/27) of the total population and made up mostly of sheep isolates (Fig. 1b). Both *nad*1 and *nad*5 central haplotype comprised isolates from both hosts but did not constitute the majority of the population (Fig. 1a, b). In addition, *nad*5 and *cox*1 network showed distinct haplotypes for sheep isolates (Fig. 1b, c). Interestingly, on analysis of the concatenated sequences of all three genes, all sheep isolates formed two distinct haplotypes (Fig. 1d).

**Table 2:** Diversity and neutrality indices for *Taenia hydatigena* populations from Nigeria based on *cox*1, *nad*1, *nad*5 and concatenated *cox*1-*nad*1-*nad*5 mitochondrial gene sequences

Feature/Index	*cox*1 (1620 bp)			*nad*1 (894 bp)			*nad*5 (1569 bp)			*cox*1-*nad*1-*nad*5 (4083 bp)		
	Sheep	Goats	Overall	Sheep	Goats	Overall	Sheep	Goats	Overall	Sheep	Goats	Overall
No. of isolates	6	18	24	8	21	29	8	19	27	6	16	22
No. of mutations	5	21	24	3	24	24	1	33	33	9	68	75
Parsimony informative sites	5	11	15	3	15	18	1	12	12	9	37	44
No. of haplotypes	2	9	10	2	10	10	2	8	9	2	7	9
Haplotype diversity (Hd)	0.600	0.889	0.906	0.536	0.867	0.867	0.536	0.766	0.835	0.600	0.858	0.900
Nucleotide diversity (π)	0.00185	0.00260	0.00264	0.00180	0.00729	0.00628	0.00034	0.00353	0.00270	0.00132	0.00401	0.00358
Tajima’s D (*P-*value)	2.07077*	− 1.21615	− 1.23884	1.60077	− 0.23673	− 0.42079	1.16650	− 1.64663	− 1.87248*	2.20374*	− 0.85760	− 1.15691
Fu’s Fs	4.007*	− 0.956	− 0.786	2.988*	0.199	0.771	0.866	0.934	0.266	5.778*	5.873*	4.945*

*Significant *P-*value (*P* < 0.05)

The observed nucleotide polymorphism between haplotypes resulted in amino acid change. Comparison of haplotype 1 with other haplotypes of each respective gene showed the following changes: *cox*1 gene, a single amino acid substitution (cNIG6: 447A-447V); *nad*1, 6 amino acid substitutions (aNIG3: 10V-10G, 74G-74S, 86V-86I, and 253V-253G; aNIG4: 199F-199L, and 227C-227R) and *nad*5, 8 amino acid substitutions (bNIG2: 75I-75V, and 159L-159S; bNIG3: 385I-385V; bNIG4: 93I-93V and 385I-385V; bNIG5: 57L-57S; bNIG6: 353I-353V; bNIG7: 234I-234T) (see Additional file 1: Tables S1–S3).

Representative haplotype sequences of *T. hydatigena nad*5, *nad*1, and *cox*1 genes can be found in GenBank under accession numbers, MN175571-MN175579, MN175580-MN175589 and MN175590-MN175599, respectively.

### Population indices

The pairwise nucleotide differences were highest for *nad*1 (0.1–2.5%), followed by *nad*5 (0.1–1.5%) and *cox*1 (0.1–0.7%). A high haplotype (Hd) and low nucleotide (π) diversity were observed for all three genes in both intermediate hosts (Table 2). Overall, Hd and π were as follows: *cox*1 (Hd = 0.906, π = 0.00264), *nad*1 (Hd = 0.867, π = 0.00628) and *nad*5 (Hd = 0.835, π = 0.00270). *Taenia hydatigena* population from sheep showed positive Tajima’s *D* for all genes and was significant for *cox*1. In contrast, isolates from goats showed negative insignificant Tajima’s *D* for all genes (Table 2). Fu’s Fs was positive for all genes except for *cox*1 isolates from goats (Table 2). Analysis of the concatenated gene sequences (*cox*1-*nad*1-*nad*5; 4083 bp), resolved most inconsistencies with an overall Hd and π of 0.900 and 0.00358, respectively, and a positively significant Tajima’s *D* (2.20374) and Fu’s Fs (5.778) for isolates of sheep origin, suggesting the possibility of a balance selection or sudden population contraction. In contrast, a negative (insignificant) Tajima’s *D* (− 0.85760) and a significantly positive Fu’s Fs (5.873) were observed for isolates from goats. Using the *cox*1, *nad*1 and *nad*5 genes, F_ST_ values for pairwise comparison between isolates from goats and sheep were as follows: *cox*1 (F_ST_ = 0.183, *P* = 0.009), *nad*1 (F_ST_ = 0.147, *P* = 0.009), *nad*5 (F_ST_ = 0.052, *P* = 0.117), and *cox*1-*nad*1-*nad*5 (F_ST_ = 0.149, *P* = 0.045) suggesting the possibility of genetically differentiated strains.

### Phylogenetic analyses

**Fig. 2:**
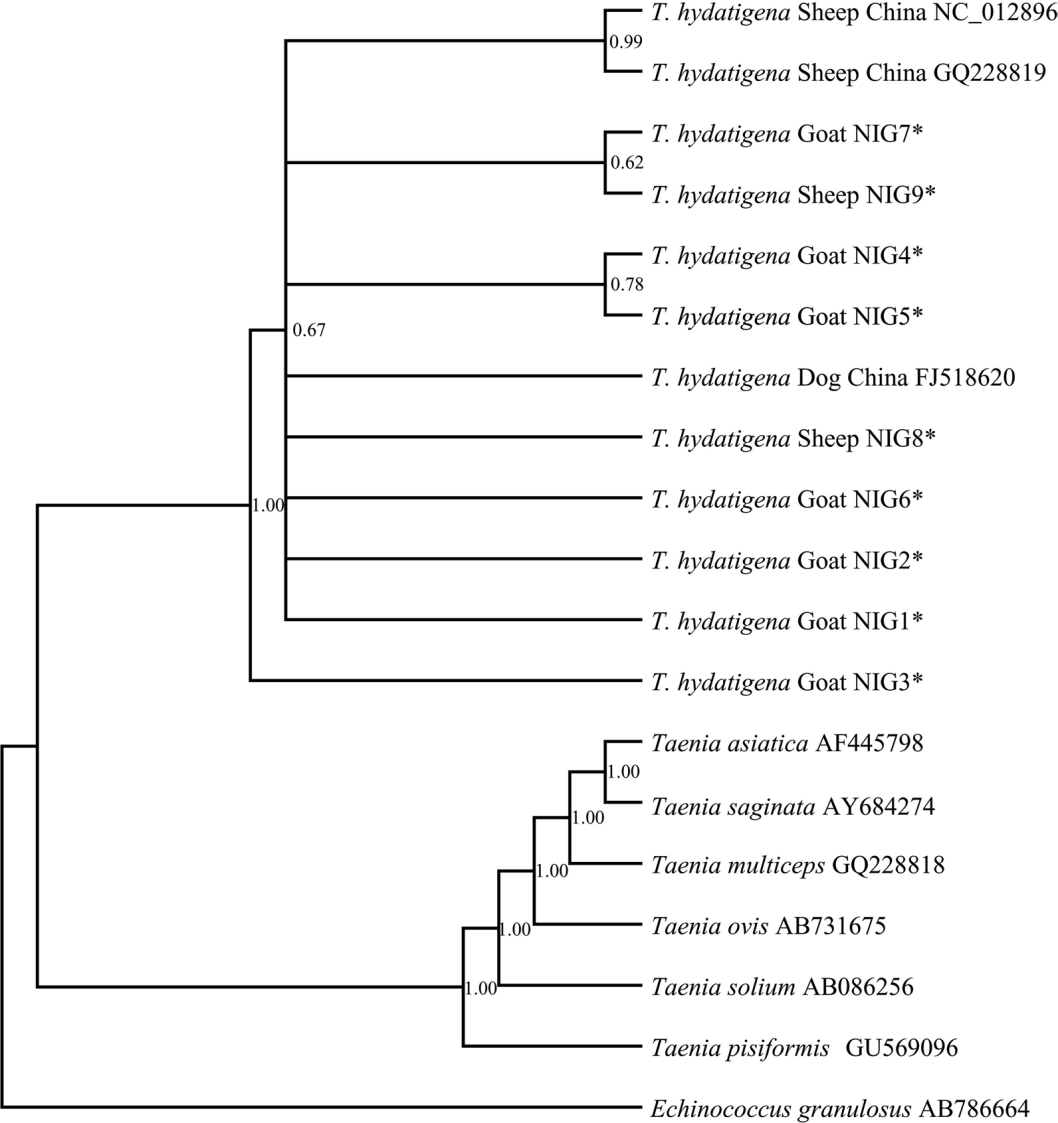
Bayesian phylogenetic relationships of the Nigerian *Taenia hydatigena* isolates based on *cox*1-*nad*1-*nad*5 (4083 bp) concatenated gene sequences. Posterior probability (pp) values are depicted at the nodes. *Echinococcus granulosus* was used as an outgroup. Asterisks (*) indicate haplotypes representing isolates from this study, GenBank: MN175594, MN175582, MN175571 (NIG1) MN175595, MN175580, MN175572 (NIG2) MN175596, MN175584, MN175573 (NIG3) MN175592, MN175585, MN175575 (NIG4) MN175599, MN175587, MN175577 (NIG5) MN175598, MN175588, MN175576 (NIG6) MN175597, MN175589, MN175571 (NIG7) MN175590, MN175581, MN175578 (NIG8) MN175591, MN175580, MN175579 (NIG9)

Phylogenetic analysis showed low posterior probability (pp) values indicating weak nodal support (< 0.80) for the different haplotypes (Fig. 2). Also, haplotype NIG3 appeared as a sister taxon to other *T. hydatigena* cluster (the same haplotype according to the median-joining network also presented at least, a 15-point mutation difference from its closest neighbour, Fig. 1d). The cluster showed three sub-clades with varying pp values of which two Chinese reference isolates formed the first sub-clade with a pp value of 0.99 compared to other sub-clades (< 0.80) which were constituted by haplotypes from Nigeria (Fig. 2).

### Comparison of Nigerian *T. hydatigena* isolates with populations from other geographical regions

**Fig. 3:**
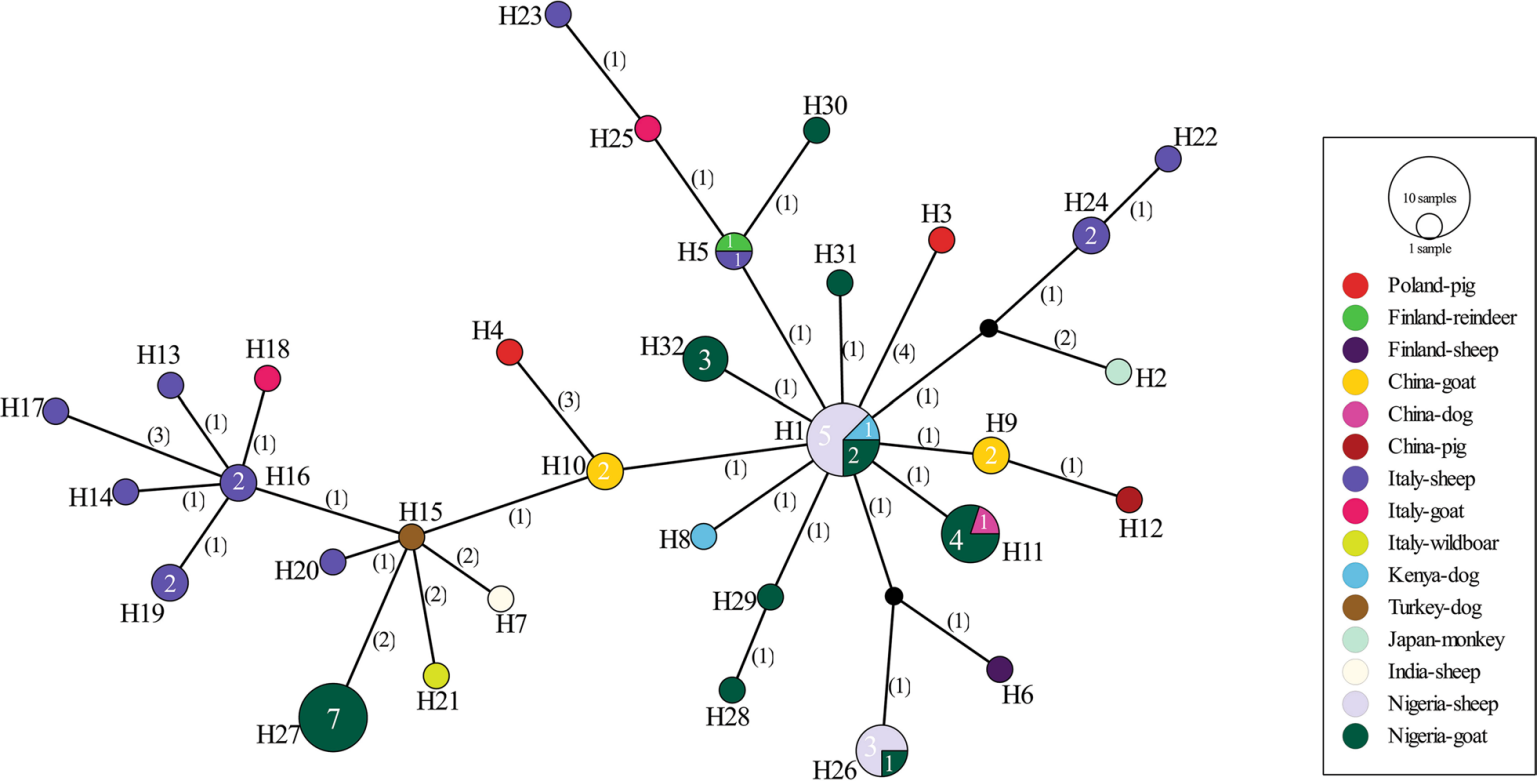
Median-joining network of *Taenia hydatigena* isolates from different hosts and geographical locations based on the NADH dehydrogenase subunit 1 (*nad*1) (435 bp) mitochondrial gene. Circle sizes are proportional to the haplotype frequencies. Numbers in parentheses represent the number of mutations. Small black circles are median vectors (i.e. hypothetical haplotypes)

Due to the scarcity of full-length mitochondrial DNA (mtDNA) data from other geographical locations as in this study, we could not employ the full-length mitochondrial genes for comparison. Nonetheless, on trimming, a final dataset of 59 *nad*1 sequences of 435 bp (including 29 sequences from this study) (Additional file 1: Table S4) was used to compare the geographical relatedness of the Nigerian isolates and those from different regions. Results of the analysis showed 32 haplotypes, a high Hd of 0.9573, and a π of 0.00853. Overall neutrality indices showed significantly negative values (Tajima’s *D* *=* − 1.81180, Fu’s Fs = − 25.300). Furthermore, the median-joining network presented two major haplogroups (Fig. 3) that comprised haplotypes from different hosts and locations (Fig. 3). In addition, F_ST_ values of *T. hydatigena* population based on pairwise comparison showed significant differences only between isolates of Nigerian and European origin (F_ST_ *=* 0.116, *P* = 0.009) and European and Asian/Middle Eastern isolates (F_ST_ = 0.088, *P* = 0.05) (Additional file 1: Table S5).

## Discussion

Despite the fact that cysticercosis due to *T. hydatigena* causes inconceivable damage in livestock production in endemic countries [1–4], the genetic population structure and the epidemiological significance of the observed genetic variation is poorly understood [4, 29]. In Nigeria, previous epidemiological surveys have shown that cysticercosis caused by *T. hydatigena* in sheep and goats could reach as high as 20–30% [10, 13] and below 2% in pigs [7], causing remarkable setbacks in livestock production [3]. Similarly, in other African countries, a number of epidemiological studies have also reported high prevalence of cysticercosis in ruminants [4, 42, 43].

Meanwhile, mtDNA has been widely employed in investigating the intraspecific variation of metazoans due to the absence of recombination, maternal inheritance, conserved structure, higher mutation rate and a relatively high evolutionary rate [44–47]. Another method for studying intraspecific variation or genetic diversity in cestodes is the use microsatellite DNA, which has been reported to be highly informative and also commonly used in genetic population studies [48–50]. However, in this study, we report the genetic diversity of *T. hydatigena* isolates from sheep and goats collected from a slaughterhouse in Nigeria based on complete sequences for three mitochondrial genes, *cox*1, *nad*1 and *nad*5, in contrast to the commonly employed partial gene sequences [4, 28, 51, 52].

Our analysis of the three mitochondrial genes revealed a considerable degree of genetic variation. Higher haplotype diversity was recorded for goat isolates and was highest for *cox*1 gene while nucleotide diversity was similarly higher in goats and highest for *nad*1 gene. Overall, the diversity and neutrality indices based on concatenation of all three genes were higher in goats and demonstrated inconsistency with population expansion which suggest the possibility of a bottleneck event in the course of evolution. However, the diversity indices of the individual genes *cox*1 and *nad*1 were comparable to estimates from Iranian and Italian (Sardinia) *T. hydatigena* populations from sheep and goats [28] but higher than Palestinian (West Bank) *T. hydatigena* populations of sheep origin [51]. In Palestine, lower prevalence and transmission rate of cysticercus tenuicollis (the larval stage of *T. hydatigena*), smaller area of Palestine, low small ruminant population (about 1.5 million) [53] as well as the prevailing management system have been suggested to influence the genetic diversity of *T. hydatigena* [28, 51]. In contrast, Iran and Sardinia (Italy) experience a higher prevalence and transmission rate of cysticercosis, higher population of small ruminants (Iran ≥ 50 million, Sardinia ≥ 3 million), with traditional farming methods still being applied in raising sheep and goats [28]. These differences could possibly have an influence on the genetic diversity of *T. hydatigena*, such that a higher prevalence in intermediate hosts, as seen in Italy and Iran, may result in multiple infections (cysts from different intermediate hosts) in definitive hosts favouring sexual reproduction and consequently higher genetic variation within the population.

Although the significant differences in the number of examined Nigerian *T. hydatigena* isolates (from both intermediate hosts) could have influenced the outcome of the neutrality and nucleotide diversity indices, the hypothetical existence of host-specific strains which has been reported cannot be completely ruled out [28, 54, 55]

Based on the individual genes, the median-joining network featured a centralized haplotype only for *nad*1 and *nad*5 genes but these did not constitute the majority of the population. On concatenation of the three genes, which is believed to be more informative than the individual genes, no centralized haplotype was found. However, two distinct haplotypes were revealed in all sheep isolates. Meanwhile, the formation of a founder haplotype (29.2% of the population) based on partial *cox*1 (324 bp) gene was reported in a pooled population of *T. hydatigena* from different countries and hosts [28]. Conversely, analysis of the *nad*1 gene in the latter study did not reveal a centralised haplotype. The same was true for Italian isolates from sheep, goats and wild boars [28]. In Palestine, the formation of a founder haplotype was also observed in sheep based on *cox*1 fragment (444 bp) and constituted about 55% of the examined population [51]. However, in a more recent investigation on the genetic diversity of *T. hydatigena* isolates from Turkish sheep and goats based on *nad*1 fragment (471 bp), no centralised haplotype was also observed [52]. Be that as it may, clarification of such discrepancies and improved phylogenetic resolution rest mostly on the analysis of longer or complete mtDNA sequences.

We observed high F_ST_ values between *T. hydatigena* populations from sheep and goats, suggesting genetic differentiation. Meanwhile, reports of host specificity have been previously documented between isolates from sheep and goats in Iran [54] and India [55], such that *T. hydatigena* metacestodes from both hosts were found to be morphologically different. Similarly, analysis of the biochemical components of cysticerci from pigs and goats also suggested genetic differences [56]. More so, pairwise comparisons in the present study between goats and sheep isolates yielded a significant F_ST_ value. This is in-line with previous F_ST_ values suggesting genetic differentiation between sheep and goat isolates from Italy, China and Greece, as well as between pig isolates from Italy, China and Poland [28], which further supports the idea of genetic distinction among *T. hydatigena* populations infecting different hosts. Then again, the observed pairwise fixation values and population indices could have been influenced by the small sample size analysed in the present study as sample size has been found to influence estimates of population indices [38].

Phylogenetic analysis of the concatenated gene sequences showed that all isolates from Nigeria were correctly identified as *T. hydatigena* as they clustered closely with other *T. hydatigena* isolates from other regions with relative distances from other *Taenia* spp. (Fig. 2). However, haplotype NIG3 which consists only of isolates from goats formed a separate clade (Fig. 2). The weak nodal support observed between Nigerian haplotypes is similar to previous observations of low posterior probability values (0.51–1.00) and bootstrap values (79%) between *T. hydatigena* haplotypes from Iran and Tanzania [4, 27].

As previously suggested [56], strain specificity in *T. hydatigena*, may present a similar feature like that of *Echinococcus* in which case, deep insight may be provided potentially on examination of longer mtDNA fragments and possibly nuclear genes of larger datasets comprising isolates from different hosts and geographical locations.

Furthermore, analysis of the trimmed *nad*1 gene sequence dataset (435 bp) from *T. hydatigena* isolates from other geographical regions and hosts revealed a population network that was consistent with population expansion based on the significant negative Tajima’s *D* and Fu’s F, indicating the presence of rare haplotypes as well as the characteristic star-shaped configuration with a centrally placed haplotype similar to previous observation by Boufana et al. [28]. In the network, some haplotypes were formed by isolates from different hosts and locations. Conversely, the distinct haplotypes of Nigerian sheep origin previously classified based on 4083 bp of mitochondrial DNA, formed a new haplotype that comprised isolates from different hosts (including Nigerian goat isolates) and locations, thus questioning the reliability of partial gene sequences in investigating the genetic population structure or resolving the phylogenetic status of *T. hydatigena*. Moreover, the pitfall of short gene sequences in inferring the phylogenetic status and the genetic diversity of cestodes has been emphasized for *Echinococcus* spp. [57]. Further, the outcome of longer DNA fragment analysed in the present study demonstrated that using either of the genes alone is insufficient to describe the intraspecific variation existing between *T. hydatigena*, as the 4083 bp of the mitochondrial genome provided a better resolution as indicated by the phylogenetic network and population indices.

The influence of geography or animal husbandry practices could not have plausibly explained the genetic differences observed between *T. hydatigena* isolates in this study. This is because small ruminants (sheep and goats) in Nigerian pastoral communities are mostly raised in the same environment and similar animal husbandry system. Moreover, each geopolitical zone of the country share similar geography (vegetation and climatic conditions), and in this study, the examined ruminants originated from the same state.

## Conclusion

The present study, to the best of our knowledge, described the genetic variation of *T. hydatigena* in Nigeria for the first time using full-length mitochondrial gene sequences and suggests the possible existence of host-specific strains. The scarcity of complete *T. hydatigena* mitochondrial *cox*1, *nad*1 and *nad*5 sequences from different geographical regions in the GenBank repository limited a wide-range comparison and interpretation. However, the results suggest that longer DNA fragments or complete mitochondrial genome analysis may provide a better resolution in our understanding of the genetic diversity of *T*. *hydatigena* and the possible epidemiological significance.

## Supplementary information

**Additional file 1: Table S1.** *Taenia hydatigena* mitochondrial *cox*1 gene nucleotide sequence polymorphism and corresponding amino acid changes among haplotypes from sheep and goats. **Table S2.** *Taenia hydatigena* mitochondrial *nad*1 gene nucleotide sequence polymorphism and corresponding amino acid changes among haplotypes from sheep and goats. **Table S3.** *Taenia hydatigena* mitochondrial *nad*5 gene nucleotide sequence polymorphism and corresponding amino acid changes among haplotypes from sheep and goats. **Table S4.** Characteristics of *Taenia hydatigena* isolates used in this study. **Table S5.** Comparison of pairwise fixation values (F_ST_) for *Taenia hydatigena* isolates from different geographical regions compared to those from Nigeria based on partial *nad*1 mitochondrial gene sequences.

## Data Availability

All data supporting the conclusions of this article are included in the article and its additional files. Representative nucleotide sequences of *cox*1, *nad*1 and *nad*5 genes from the present study are available in the GenBank database under the accession numbers MN175571-MN175599.

## Abbreviations

PCR: polymerase chain reaction
pp: posterior probability
*cox*1: cytochrome *c* oxidase subunit 1 gene
*nad*1: NADH dehydrogenase subunit 1 gene
*nad*5: NADH dehydrogenase subunit 5 gene
mtDNA: mitochondrial DNA
MCMC: Markov Chain Monte Carlo
Hd: haplotype diversity
