# Correction to: First molecular description, phylogeny and genetic variation of *Taenia hydatigena* from Nigerian sheep and goats based on three mitochondrial genes

**DOI:** 10.1186/s13071-019-3807-y

**Published:** 2019-11-21

**Authors:** John A. Ohiolei, Joshua Luka, Guo-Qiang Zhu, Hong-Bin Yan, Li Li, Abdullahi A. Magaji, Mughees A. Alvi, Yan-Tao Wu, Jian-Qiu Li, Bao-Quan Fu, Wan-Zhong Jia

**Affiliations:** 1 State Key Laboratory of Veterinary Etiological Biology/National Professional Laboratory of Animal Hydatidosis/Key Laboratory of Veterinary Parasitology of Gansu Province, Lanzhou Veterinary Research Institute, Chinese Academy of Agricultural Sciences, Lanzhou, 730046 People’s Republic of China; 2 Department of Veterinary Parasitology and Entomology, Faculty of Veterinary Medicine, University of Maiduguri, Maiduguri, Nigeria; 3 Department of Veterinary Public Health and Preventive Medicine, Faculty of Veterinary Medicine, Usman Danfodiyo University, Sokoto, Nigeria

## Correction to: Parasites Vectors (2019) 12:520 10.1186/s13071-019-3780-5

Following publication of the original article [1], the authors have flagged that the information in the legend of Fig. 1 is detailed in the wrong order.

Please note that the (correct) legend for Fig. 1 is:

**Fig. 1.** Median-joining networks of *Taenia hydatigena* isolates from Nigeria based on complete *nad*1 (**a**) *nad*5 (**b**) *cox*1 (**c**) and concatenated *cox*1-*nad*1-*nad*5 gene sequences (**d**). Circle sizes are proportional to the haplotype frequencies. Numbers in parentheses represent the number of mutations. Small black circles are median vectors (i.e. hypothetical haplotypes).

The authors apologize for any inconvenience caused.
