# Supplementary material for: First molecular description, phylogeny and genetic variation of Taenia hydatigena from Nigerian sheep and goats based on three mitochondrial genes
Source: Parasit Vectors. 2019 Nov 5;12:520. doi: 10.1186/s13071-019-3780-5 (PMC6833231; doi:10.1186/s13071-019-3780-5)
Supplement: Supplementary file 1 — Additional file 1: Table S1. Taenia hydatigena mitochondrial cox1 gene nucleotide sequence polymorphism and corresponding amino acid changes among haplotypes from sheep and goats. Table S2. Taenia hydatigena mitochondrial nad1 gene nucleotide sequence polymorphism and corresponding amino acid changes among haplotypes from sheep and goats. Table S3. Taenia hydatigena mitochondrial nad5 gene nucleotide sequence polymorphism and corresponding amino acid changes among haplotypes from sheep and goats. Table S4. Characteristics of Taenia hydatigena isolates used in this study. Table S5. Comparison of pairwise fixation values (FST) for Taenia hydatigena isolates from different geographical regions compared to those from Nigeria based on partial nad1 mitochondrial gene sequences. [file 13071_2019_3780_MOESM1_ESM.docx]

**Additional file 1: Table S1.** *Taenia hydatigena* mitochondrial *cox*1 gene nucleotide sequence polymorphism and corresponding amino acid changes among haplotypes from sheep and goats.

Table S1a: *cox*1 mutation sites

| Haplotype | *cox*1 DNA mutation site | | | | | | | | | | | | | | | | | | | | | | | |
| --- | --- | --- | --- | --- | --- | --- | --- | --- | --- | --- | --- | --- | --- | --- | --- | --- | --- | --- | --- | --- | --- | --- | --- | --- |
|  | 159 | 225 | 261 | 375 | 396 | 564 | 586 | 750 | 786 | 807 | 837 | 924 | 987 | 1056 | 1155 | 1257 | 1290 | 1314 | 1329 | 1340 | 1374 | 1422 | 1545 | 1554 |
| cNIG1 | C | C | C | T | T | T | T | G | C | A | A | C | C | T | T | C | T | C | G | C | T | A | T | T |
| cNIG2 | T |  |  |  |  |  |  | A |  |  |  | T |  |  |  |  |  |  |  |  | C |  | C |  |
| cNIG3 | T |  | T |  |  |  |  | A |  |  |  |  |  |  |  |  |  |  | A |  |  |  | C |  |
| cNIG4 | T |  |  |  | C |  |  | A |  |  |  |  |  | C |  |  |  |  |  |  |  |  | C |  |
| cNIG5 | T |  |  |  |  |  | C | A |  |  |  |  |  |  |  |  |  |  |  |  |  |  | C |  |
| cNIG6 | T |  |  |  |  |  |  | A |  |  |  |  |  |  |  | T |  |  |  | T |  | G | C | C |
| cNIG7 | T |  |  | C |  | C |  | A | T | G |  |  |  |  |  |  |  |  |  |  |  |  | C |  |
| cNIG8 | T | T |  |  |  |  |  | A |  |  |  | T |  |  |  |  |  |  |  |  |  |  | C |  |
| cNIG9 | T |  |  |  |  |  |  | A |  |  |  |  | T |  |  |  |  |  |  |  |  |  | C |  |
| cNIG10 | T |  |  |  |  |  |  | A |  |  | T |  |  |  | C |  | C | T |  |  |  |  | C |  |

Table S1b: *cox*1 amino acid substitution

| Haplotype | *cox*1 amino acid substitution |
| --- | --- |
|  | 447 |
| cNIG1 | A |
| cNIG2 |  |
| cNIG3 |  |
| cNIG4 |  |
| cNIG5 |  |
| cNIG6 | V |
| cNIG7 |  |
| cNIG8 |  |
| cNIG9 |  |
| cNIG10 |  |

**Additional file 1: Table S2.** *Taenia hydatigena* mitochondrial *nad*1 gene nucleotide sequence polymorphism and corresponding amino acid changes among haplotypes from sheep and goats.

Table S2a: *nad*1 mutation sites

| Haplotype | *nad*1 DNA mutation site | | | | | | | | | | | | | | | | | | | | | | | |
| --- | --- | --- | --- | --- | --- | --- | --- | --- | --- | --- | --- | --- | --- | --- | --- | --- | --- | --- | --- | --- | --- | --- | --- | --- |
|  | 29 | 78 | 207 | 213 | 220 | 222 | 241 | 246 | 256 | 297 | 519 | 528 | 543 | 595 | 603 | 666 | 678 | 679 | 750 | 758 | 760 | 834 | 852 | 882 |
| aNIG1 | T | C | T | A | G | T | C | C | G | C | G | A | A | T | C | T | A | T | G | T | T | A | T | G |
| aNIG2 |  |  |  | G |  |  |  | T |  |  |  |  |  |  |  |  |  |  |  |  |  | T |  |  |
| aNIG3 | G | T |  |  | A |  | T |  | A | T |  |  |  |  |  |  |  |  |  | G |  |  |  |  |
| aNIG4 |  |  |  |  |  |  |  |  |  |  |  |  | G | C |  |  |  | C |  |  |  |  |  |  |
| aNIG5 |  |  |  |  |  |  |  |  |  |  |  |  |  |  | T | A |  |  |  |  |  |  | C | T |
| aNIG6 |  |  |  |  |  |  |  |  |  |  |  |  | G |  |  |  |  |  | A |  |  |  |  |  |
| aNIG7 |  |  |  |  |  |  |  |  |  |  |  |  |  |  | T | A |  |  |  |  |  |  | C |  |
| aNIG8 |  |  | A |  |  | C |  |  |  |  |  |  |  |  |  |  |  |  |  |  |  |  |  |  |
| aNIG9 |  |  |  |  |  |  |  |  |  |  | A |  |  |  |  |  |  |  |  |  |  |  |  |  |
| aNIG10 |  |  |  |  |  |  |  |  |  |  |  | G |  |  |  |  | T |  |  |  | C |  |  | A |

Table S2b: *nad*1 amino acid substitution

| Haplotype | *nad*1 amino acid substitution | | | | | |
| --- | --- | --- | --- | --- | --- | --- |
|  | 10 | 74 | 86 | 199 | 227 | 253 |
| aNIG1 | V | G | V | F | C | V |
| aNIG2 |  |  |  |  |  |  |
| aNIG3 | G | S | I |  |  | G |
| aNIG4 |  |  |  | L | R |  |
| aNIG5 |  |  |  |  |  |  |
| aNIG6 |  |  |  |  |  |  |
| aNIG7 |  |  |  |  |  |  |
| aNIG8 |  |  |  |  |  |  |
| aNIG9 |  |  |  |  |  |  |
| aNIG10 |  |  |  |  |  |  |

**Additional file 1: Table S3.** *Taenia hydatigena* mitochondrial *nad*5 gene nucleotide sequence polymorphism and corresponding amino acid changes among haplotypes from sheep and goats.

Table S3a: *nad*5 mutation sites

| Haplotype | | *nad*5 DNA mutation site | | | | | | | | | | | | | | | | | | | | | | | | | | | | | | | | | |
| --- | --- | --- | --- | --- | --- | --- | --- | --- | --- | --- | --- | --- | --- | --- | --- | --- | --- | --- | --- | --- | --- | --- | --- | --- | --- | --- | --- | --- | --- | --- | --- | --- | --- | --- | --- |
|  | 108 | | 126 | 153 | 170 | 223 | 264 | 277 | 300 | 426 | 462 | 476 | 480 | 504 | 525 | 555 | 570 | 606 | 660 | 685 | 701 | 759 | 811 | 843 | 990 | 1057 | 1095 | 1153 | 1161 | 1164 | 1296 | 1320 | 1362 | 1494 |  |
| bNIG1 | G | | T | A | T | A | T | A | T | G | G | T | A | T | G | T | C | A | T | T | T | G | T | G | A | A | G | A | A | A | T | C | A | A |  |
| bNIG2 |  | |  |  |  | G |  |  |  |  |  | C |  |  |  |  |  |  |  |  |  | A |  |  | G |  | A |  |  |  |  |  |  |  |  |
| bNIG3 |  | |  |  |  |  |  |  |  | A |  |  | G |  |  |  |  |  |  |  |  |  | C |  | G |  |  | G |  |  |  |  | G |  |  |
| bNIG4 |  | |  |  |  |  |  | G |  | A |  |  |  |  |  | C |  |  |  |  |  |  |  | A | G |  |  | G |  |  |  |  | G |  |  |
| bNIG5 | A | |  |  | C |  | C |  |  |  |  |  |  | C |  |  |  | G |  |  |  |  |  |  | G |  |  |  |  | G |  |  |  |  |  |
| bNIG6 |  | |  |  |  |  |  |  |  |  |  |  |  |  | A |  | T |  |  | C |  |  |  |  | G | G |  |  |  |  |  |  |  | G |  |
| bNIG7 | A | | C | G |  |  |  |  | C |  | T |  |  | C |  |  |  |  | C |  | C |  |  |  | G |  |  |  | G |  | C | T |  |  |  |
| bNIG8 | A | |  |  |  |  |  |  |  |  |  |  |  |  |  |  |  |  |  |  |  |  |  |  | G |  |  |  |  |  |  |  |  |  |  |
| bNIG9 |  | |  |  |  |  |  |  |  |  |  |  |  |  |  |  |  |  |  |  |  |  |  |  | G |  |  |  |  |  |  |  |  |  |  |

Table S3b: *nad*5 amino acid substitution

| Haplotype | *nad*5 amino acid substitution | | | | | | |
| --- | --- | --- | --- | --- | --- | --- | --- |
|  | 57 | 75 | 93 | 159 | 234 | 353 | 385 |
| bNIG1 | L | I | I | L | I | I | I |
| bNIG2 |  | V |  | S |  |  |  |
| bNIG3 |  |  |  |  |  |  | V |
| bNIG4 |  |  | V |  |  |  | V |
| bNIG5 | S |  |  |  |  |  |  |
| bNIG6 |  |  |  |  |  | V |  |
| bNIG7 |  |  |  |  | T |  |  |
| bNIG8 |  |  |  |  |  |  |  |
| bNIG9 |  |  |  |  |  |  |  |

**Additional file 1**: **Table S4.** Characteristics of *Taenia hydatigena* isolates used in this study.

| Origin | Host | No of isolates | Accession number |
| --- | --- | --- | --- |
| China | Goat | 3 | JN831270, JN831279, JN831281 |
|  | Dog | 1 | HQ204204 |
|  | Pig | 1 | JN831286 |
|  |  |  |  |
| Finland | Reindeer | 1 | EU544605 |
|  | Sheep | 1 | EU544604 |
| India | Sheep | 1 | DQ995654 |
| Italy | Sheep | 13 | KT372532- KT372534, KT372536, KT372537, KT372539, KT372540, KT372542, KT372543, KT372546- KT372548, KT372550 |
|  | Goat | 2 | KT372535, KT372549 |
|  | Wild boar | 1 | KT372541 |
| Japan | Monkey | 1 | AB304465 |
| Kenya | Dog | 2 | AM503332, AM503334 |
| Nigeria | Sheep | 8 | This study |
|  | Goat | 21 | This study |
|  |  |  |  |
| Poland | Pig | 2 | AJ277408, AJ277409 |
| Turkey | Dog | 1 | KC876043 |

**Additional file 1: Table S5**. Comparison of pairwise fixation values (F_ST_) for *Taenia hydatigena* isolates from different geographical regions compared to those from Nigeria based on partial *nad*1 mitochondrial gene sequences.

|  | Nigeria | East Africa | Asia & Middle East | Europe |
| --- | --- | --- | --- | --- |
| Nigeria (29) | - |  |  |  |
| East Africa (2) | -0.071 (*p*= 0.685) | - |  |  |
| Asia & Middle East (7) | 0.052 ( *p*= 0.162) | -0.105 ( *p*= 0.712) | - |  |
| Europe (21) | 0.116 ( *p*= 0.009) | -0.008 ( *p*= 0.486) | 0.088 ( *p*= 0.05) | - |
